# Self-reported physical activity and different cardiovascular diseases—Results from updated measurements over 40 years

**DOI:** 10.1371/journal.pone.0269402

**Published:** 2022-06-03

**Authors:** Lars Lind, Björn Zethelius, Liisa Byberg

**Affiliations:** 1 Department of Medical Sciences, Faculty of Medicine, Uppsala University, Uppsala, Sweden; 2 Department of Public Health and Caring Sciences/Geriatrics, Faculty of Medicine, Uppsala University, Uppsala, Sweden; 3 Department of Surgical Sciences/Medical Epidemiology, Faculty of Medicine, Uppsala University, Uppsala, Sweden; Albert Einstein College of Medicine, UNITED STATES

## Abstract

**Background:**

Self-reported leisure-time physical activity (PA) has previously been linked to risk of cardiovascular disease (CVD). We now aim to investigate the strength of associations between PA and different CVDs and how the risk varies with age.

**Methods:**

PA and traditional CV risk factors assessed by a questionnaire on a four-level scale in 2,175 men at age 50 years in the ULSAM study. Examinations were thereafter repeated at ages 60, 70, and 77.

**Results:**

During 40 years follow-up, 883 individuals experienced a CVD (myocardial infarction, stroke, or heart failure). Using data from all four examinations, a graded reduction in risk of incident CVD was seen with increasing PA (HR 0.84, 95%CI; 0.77–0.93, *p* = 0.001 for trend test). PA was related to myocardial infarction (HR 0.84, 95%CI; 0.74–0.95, 490 cases), heart failure (HR 0.79, 95%CI; 0.68–0.91, 356 cases), but only of borderline significance vs ischemic stroke (HR 0.85, 95%CI; 0.73–1.00, 315 cases) when the CVDs were analyzed separately. Adjusting for traditional CV risk factors attenuated all relationships between PA and incident CVD, and PA did not improve discrimination of CVD when added on top of risk factors. When 10-year risk was calculated from each examination, age 70 was the time-point when PA was most closely related to incident CVD.

**Conclusion:**

Leisure-time physical activity is related to future CVD. This was most evident at 70 years of age. If a causal relationship between self-reported PA and CVD exists, this relationship might to a major degree be mediated by traditional risk factors.

## Introduction

A great number of prospective studies have been conducted regarding the role of self-reported leisure-time physical activity (PA) and future cardiovascular disease (CVD). A number of meta-analyses have presented summaries of findings from these original studies and uniformly shown that a high PA is associated with a reduced risk of myocardial infarction/coronary heart disease [1, 2], stroke [1] and heart failure [3, 4]. Of those meta-analyses, only one compared these three major CVDs and found very similar risk ratios when inactive subjects were compared to those achieving current PA recommendations (11.25 metabolic equivalent of task (MET) h/week), being 0.80 for coronary heart disease, 0.81 for heart failure, and 0.82 for stroke, although the number of studies underlying those risks estimates differed between the CVDs [5]. Therefore, it would be of value to study the impact of PA on the three major CVDs in the same sample.

A second research question of interest regarding PA and future CVD is if a high PA is associated with a lower risk of CVD at all ages. We have recently investigated this issue regarding traditional CVD risk factors and the metabolic syndrome, and found a generally declining trend in the strength of the risk factors by ageing, although some risk factors still were powerful as risk factors also at high age [6, 7].

Since it is known from intervention studies that PA could have positive effects on a number of traditional CVD risk factors, such as a better glucose control [8], improved lipid levels [9], and a lower blood pressure [10], a third research question of interest is to study how the risk estimates for CVDs change after adjustment for these risk factors.

In the Uppsala Longitudinal Study of Adult Men (ULSAM) study, we have collected serial determinations of PA at four occasions during 27 years (ages 50, 60, 70 and 77) together with a follow-up of CVD over four decades. We therefore used that cohort study for three purposes. First, to study if the relationships between PA and incident cases of the three major CVDs differed using all four determinations of PA during the follow-up period. Second, to study if the relationship between PA and incident CVD differed depending on at which age PA was assessed. Third, to investigate how the relationship between PA and incident CVD changed following adjustment for traditional CVD risk factors.

## Methods

In 1970–74, 2,322 men all aged 50 years living in the city of Uppsala, Sweden, were investigated as part of the Uppsala Longitudinal Study of Adult Men (ULSAM, https://www.pubcare.uu.se/ulsam/). Of the invited individuals, 82% accepted to participate.

This cohort have since then been investigated at ages 60, 70 and 77 years.

The study was approved by the Regional Ethics Committee of Uppsala University and all study subjects had given their written informed consent to participate.

### Traditional risk factors

The baseline examination of ULSAM in the early seventies when participants were 50 years old has been described in detail previously [11]. Fasting blood samples were drawn in the morning after an overnight fast. Serum levels of cholesterol, triglycerides, and HDL were assayed by enzymatic techniques. Friedewald’s formula was used to calculate LDL-cholesterol. Moreover, fasting plasma glucose was measured using an oxidase method. Supine systolic and diastolic blood pressures were measured twice in the right arm after 10 minutes rest, and means were calculated. Data on smoking status at baseline was based on a questionnaire. BMI was calculated by weight/squared height.

PA was at each examination assessed by a questionnaire and the sample was divided into four groups: 1. Mainly sedentary behavior. 2. Walking or cycling for pleasure. 3. Recreational sports or heavy gardening at least 3 hours every week. 4. Regularly engage in hard physical training.

### CVD diagnosis

Data on cause-of-death and hospitalizations were retrieved from the Swedish Cause of Death Register and the Swedish Hospital Discharge Register, respectively. These registries, held by the Swedish National Board of Health and Wellbeing, is mandatory to report to from the Swedish health care system. The four major cardiovascular diseases were defined as; acute myocardial infarction (International Classification of Diseases [ICD-8] code 410, ICD- 9 code 410, or ICD-10 code I20), ischemic stroke (ICD-8 codes 431, 433–436, ICD-9 code 431, 433–436, ICD-10 code I63-I66), heart failure (ICD-8 codes 427.00, 427.10, 428.99, ICD-9, 428, and ICD-10 code I50), as well as hypertensive heart disease with heart failure (I11.0 [ICD-10]). The accuracy of myocardial infarction, stroke and atrial fibrillation in the Swedish registers have been deemed of high quality [12]. As the heart failure diagnosis has shown less validity, we performed additional chart review based validation of heart failure events as previously described [13]. There was no loss of follow-up. The baseline examination was performed in 1970–1974 and data on cause-of-death and hospitalizations were obtained to December 31^st^, 2014, giving four decades of follow-up.

### Statistics

The analyses were carried out using Cox proportional hazard models. Time at risk was calculated from the date of examination until date of CVD end-point, date of death, or end of follow-up (31 December, 2014), whichever occurred first. In one set of models, a trend test for PA was carried out and in another set of models PA was treated as an ordinal variable with the sedentary group as reference and the other groups being compared to that reference.

For the first research question, we applied a model with time-updated independent variables (in this case PA) for the four examination cycles for a combined CVD end-point (myocardial infarction OR ischemic stroke OR heart failure) followed by separate analyses of the three CVDs. Age was used as the time-scale.

For the second research question, we added time-updated information on the traditional CV risk factors systolic blood pressure, LDL- and HDL-cholesterol, BMI, diabetes, and smoking to the model. We also investigated how much PA added to the discrimination of CVD obtained by the traditional CV risk factors by using logistic regression and C-statistics.

For the third research question, we studied 10-year follow-ups from each examination cycle (50, 60, 70, and 77 year) and used the information on PA at each examination as the baseline exposure variable. Study participants with prevalent CVD at the time of the baseline were excluded from the analyses.

Stata16 (Stata inc, College Station, TX) was used for the calculations.

## Results

**Fig 1:**
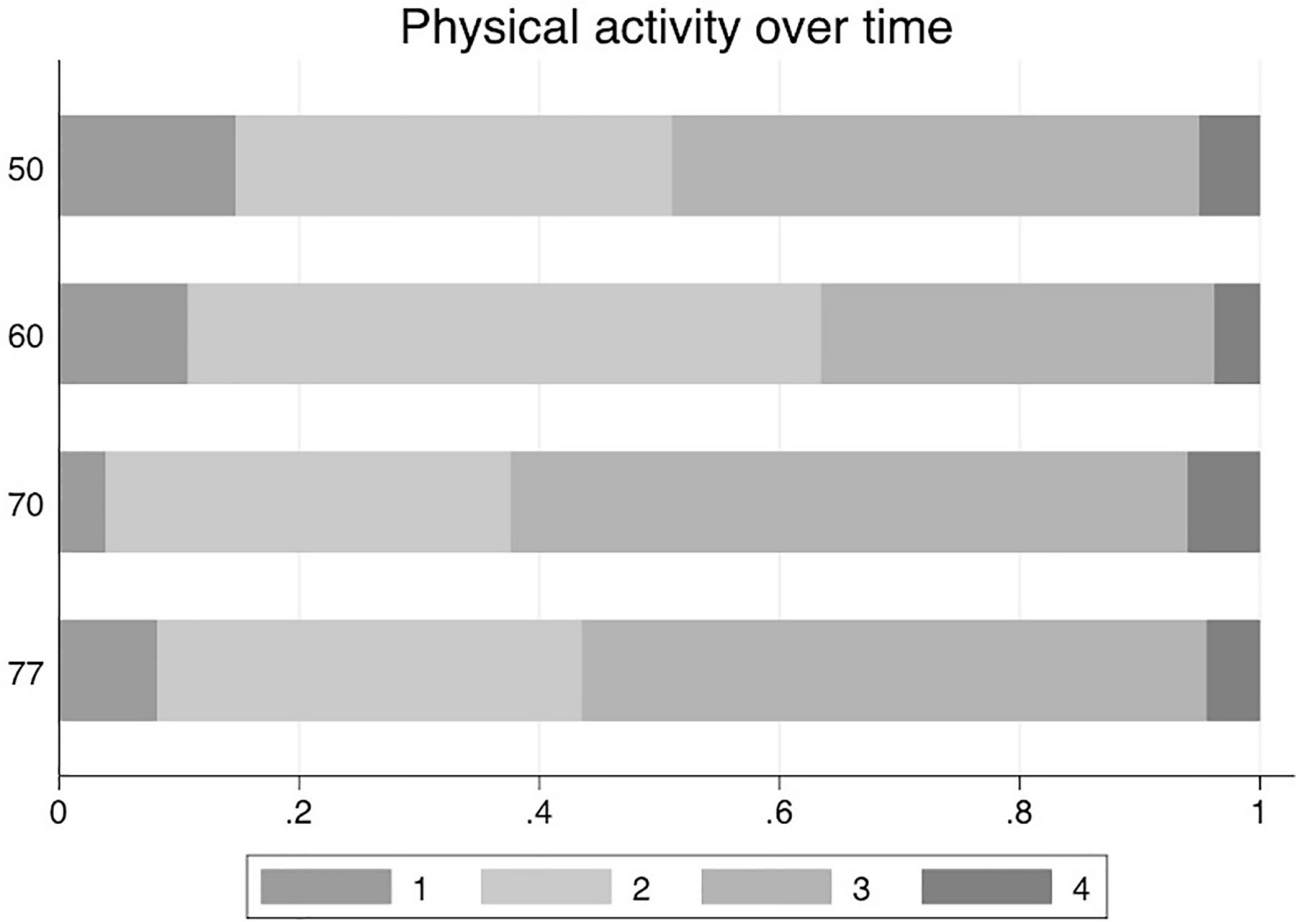
Proportions of the four levels of physical activity (PA) groups at the four different investigation cycles in the ULSAM-cohorts.

Proportions of the four categories of PA at the four different examination cycles are presented in Fig 1. Basic characteristics at the four different examinations are given in Table 1.

**Table 1:** Basic characteristics (mean (SD)) at the four examination cycles in the ULSAM-cohort for participants with full information on physical activity.

	50 years (n = 2,175)	60 years (n = 1,616)	70 years (n = 1,092)	77 years (n = 762)
LDL-cholesterol (mmol/l)	5.2 (1.1)	4.4 (0.6)	3.8 (0.9)	3.4 (0.8)
HDL-cholesterol (mmol/l)	1.3 (0.3)	1.3 (0.2)	1.3 (0.3)	1.3 (0.3)
BMI (kg/m^2^)	24.9 (3.1)	25.4 (3.2)	26.1 (3.3)	26.2 (3.4)
Systolic blood pressure (mmHg)	133 (17)	142 (19)	146 (18)	150 (20)
Diabetes (%)	4	5	11	14
Smoking (%)	51	31	17	8

### PA vs combined end-point and different incident CVDs

During a median follow-up period of 27.3 years (maximum 42.3 years), 883 events occurred regarding the combined CVD end-point during 57,827 person-year at risk (PYAR). The corresponding number of events were 490 for myocardial infarction, 315 for ischemic stroke and 356 for heart failure.

**Fig 2:**
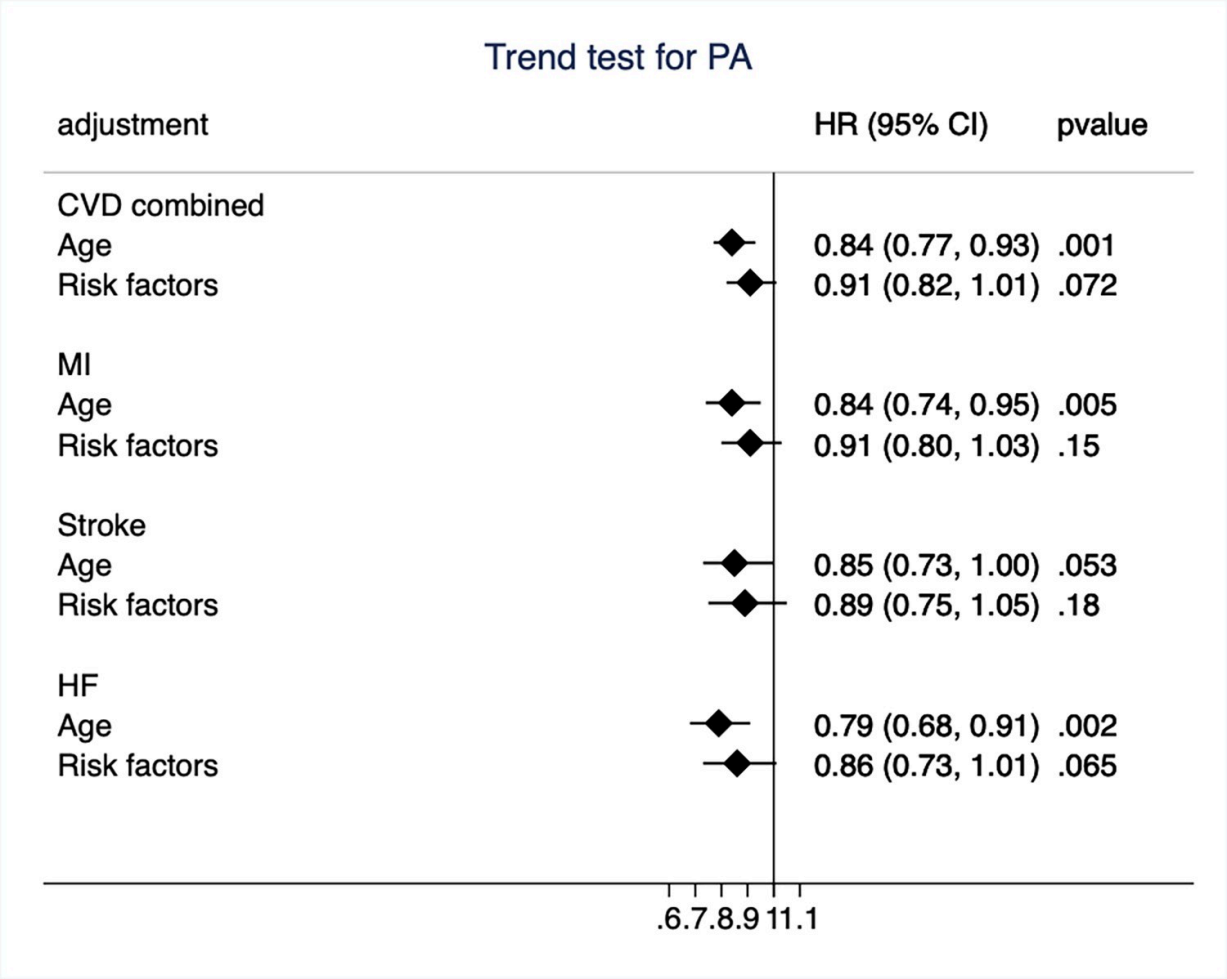
Hazard ratio (HR) and 95%CI for the trend test of physical activity (PA) for the combined end-point of cardiovascular disease (CVD) and the three CVD-components separately in models adjusted for age only and also for traditional risk factors. MI, myocardial infarction; HF, heart failure.

When we used a trend test for time-updated information on PA, it could be seen in Fig 2 that the HR for the combined end-point was 0.84 (95%CI 0.77–0.93, *p* = 0.001) for the age-adjusted analysis. The HR for the three CVDs included in the combined end-point ranged from 0.79 to 0.85 (see Fig 2 for details).

**Fig 3:**
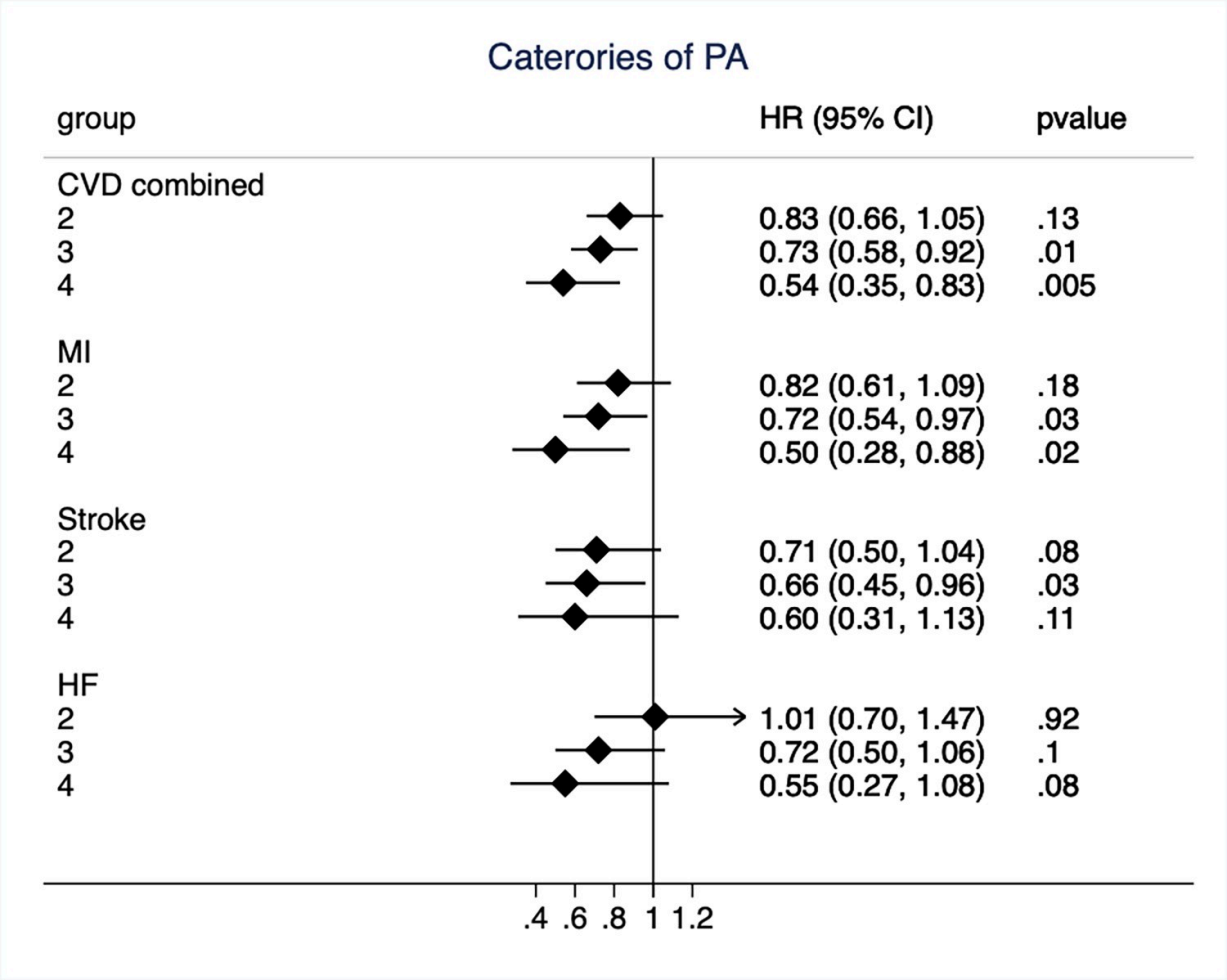
Hazard ratio (HR) and 95%CI for of physical activity (PA) when groups with PA-level 2 to 4 were compared to the reference PA-group one (sedentary) for the combined end-point of cardiovascular disease (CVD) and the three CVD-components separately in models adjusted for age only. MI, myocardial infarction; HF, heart failure.

A similar picture emerged when the three highest categories of PA were compared to the lowest category (sedentary, as presented in Fig 3), also using time-updated information on PA. A graded risk reduction was seen with higher PA category for the combined end-point, as well as the three separate outcomes. For the two highest PA categories, the risk reduction was significant when compared to the lowest category. The HRs for the highest category for the outcomes ranged from 0.50 to 0.60.

### Effect of adjustment for traditional CV risk factors

In time-updated analyses, adjustment for CVD risk factors markedly attenuated the relationships between PA and incident CVD, regardless if a trend-test or a test of the categories for PA was used and were in most cases not significant following such adjustment (see Fig 2).

When we compared the AUC curve for the traditional risk factors regarding the combined end-point vs if PA (trend) was added to the model, no improvement in C-statistics was seen (AUC 0.642 (95%CI 0.6215–0.6633) for traditional risk factors and 0.642 (95%CI 0.6219–0.6637) when PA was added to the risk factors, *p* = 0.77).

### PA measured at different time-points/examination cycles during ageing/cohort follow-up

**Fig 4:**
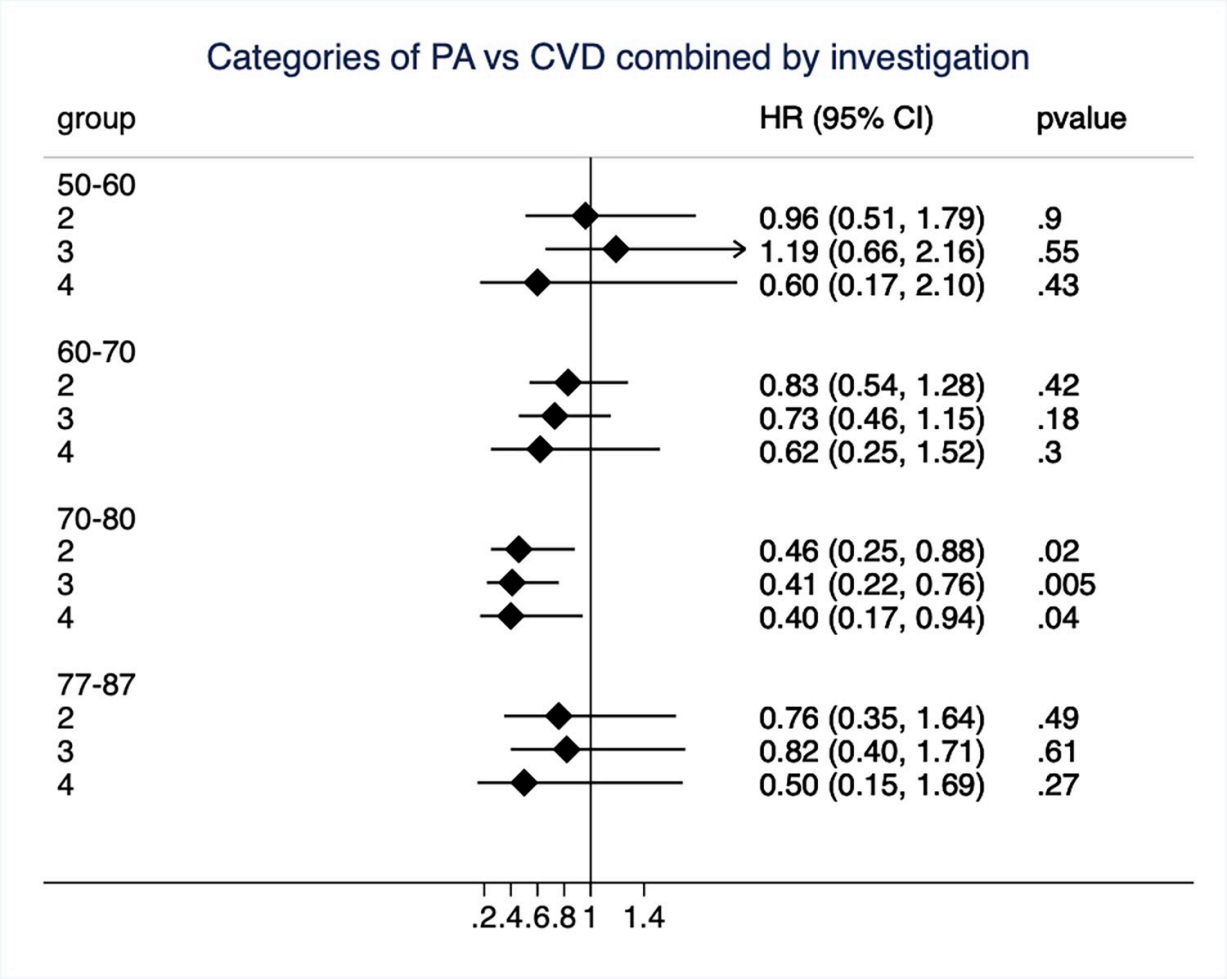
Hazard ratio (HR) and 95%CI for of physical activity (PA) when groups 2 to 4 were compared to the reference group one (sedentary) for the combined end-point of cardiovascular disease (CVD) when the 10-year risk was calculated among men free of previous CVD from each of the four investigation cycles in the ULSAM-cohort (performed at ages 50, 60, 70 and 77 years). MI, myocardial infarction; HF, heart failure.

In Fig 4, the relationships between PA at the four different examinations and incident CVD (combined end-point) during 10 years follow-up up from the respective examination are shown. The number of incident cases during the four follow-up periods were 100 during the 50-60-year period, 206 during the 60-70-year period, 192 during the 70-80-year period and 128 cases during the 77-87-year period. The only time-period that showed a clear association between PA and 10-year incident CVD was the 70 to 77-year period.

When we compared the effect of adjustment for risk factors vs the crude analysis regarding incident CVD at each follow-up period (using trend test), it was found that the adjustment increased the HR in a similar fashion at all follow-up periods, except the 50–60 period (see Table 2 for details).

**Table 2:** Hazard ratio (HR) and 95%CI of physical activity (PA, trend test) for the combined end-point of cardiovascular disease (CVD) when the 10-year risk was calculated among men free of previous CVD from each of the four investigation cycles in the ULSAM-cohort (performed at ages 50, 60, 70 and 77 years). Both the crude analysis and the analysis adjusted for traditional risk factors are shown.

Follow-up	Adjustment	HR	95%CI	p-value
50–60	Crude model	1.03	0.81–1.32	0.78
	Risk factors	1.28	0.97–1.70	0.07
60–70	Crude model	0.86	0.70–1.04	0.13
	Risk factors	0.93	0.75–1.14	0.48
70–80	Crude model	0.79	0.64–0.98	0.03
	Risk factors	0.84	0.67–1.04	0.11
77–87	Crude model	0.87	0.68–1.09	0.23
	Risk factors	1.02	0.78–1.34	0.85

## Discussion

The present study using data from four examinations during four decades of follow-up showed that PA was associated with incident CVD in an inverse fashion. Adjustment for traditional CV risk factors attenuated these relationships. The most powerful association between PA and 10-year incident CVD was seen in the 70–80 age follow-up period.

### Comparison with the literature

Multiple observational studies have demonstrated relationships between PA and incident CVD [1–5]. Since increased PA has been shown to influence the major CV risk factors in a positive fashion in intervention studies [8–10], it is assumed that the inverse relationship between PA and CVD is causal, although only a single community-based intervention study supports this [14].

The present analysis has the advantage against most other studies in this field that we by the long follow-up period (four decades) and repeated assessments of PA could evaluate the effect of PA during a major part of the adult life-span. We could also compare the risk estimates for the three major CVDs with a good power.

A meta-analysis comparing the effect of PA on different CVDs came to the conclusion that the risk estimates for myocardial infarction, heart failure and stroke were strikingly similar, ranging from 0.80 to 0.82 for the three CVDs [5]. However, the number of studies contributing to those estimates were different for the three CVDs, which makes it less valid to draw the conclusion that associations between PA and different CVDs are almost identical. In the present study, we could confirm that PA was related in an inverse fashion to the three main CVDs.

Given the effect of PA on major CV risk factors [8–10] it is not clear if any assumed positive effect of PA on future CVD is mediated by its effects on the risk factors. When we added the risk factors as co-variates in the models, a marked attenuation of the estimates for PA regarding future CVDs were noted and these relationships were in most cases not significant following this adjustment. This could be interpreted in two ways. Either as if the risk factors are mediators in the PA vs CVD relationship, or as if the risk factors are confounders effecting both PA and CVD risk. It is quite plausible that certain risk factors, like obesity, diabetes, and smoking, well could influence the ability or wish to be physically active.

Thus, it is hard to evaluate the role of traditional CV risk factors in this respect, but a formal discrimination analysis showed that adding PA to the set of traditional risk factors did not improve discrimination as assessed by C-statistics.

The impact of many of the traditional CV risk factors decline by ageing. This was however not the case in the present study in which we divided the follow-up period in 10-year periods with a new assessment of PA for each period. Contrary to what is seen for blood pressure or the metabolic syndrome [6, 7], the largest effect was not seen in the youngest subjects (in this case at 50 years). The largest impact was seen during the 70 to 80-years follow-up period. This might have several explanations. First, the number of CVD cases occurring between age 50 and 60 were rather few (n = 100), which might have resulted in uncertain estimates. Second, as could be seen in Fig 1, the proportion of subjects being sedentary were lower at age 70 compared to age 50, suggesting that many subjects have increased their PA following retirement (being 65 years in Sweden at that time), as also seen in another publication from this cohort [15]. Third, the level of PA is determined by a combination of aspiration and ability to perform exercise. At age 70, a number of conditions might have emerged, like osteoarthritis or other limiting diseases, that could limit the ability to perform exercise although the aspiration is present. Since concomitant diseases well could play a role in the development of other diseases, the ability is a component of PA that is assessed at age 70, but possibly not at age 50.

In a case where an exposure (PA in this case) could affect a confounder measured at a later occasion (risk factors), marginal structural models is the method of choice. In this rather complicated situation with four updates and varying number of observations at each update, this analysis was technically hard to perform. We therefore chose to use a model with time-updated covariates in combination with a sensitivity analysis in which we compared the effect of adjustment for risk factors using 4 different 10-year follow-ups. It was found that the risk factors induced a similar attenuation of the HR in the 60–70, 70–80 and 77–87 follow-ups as in the time-updated analysis. It is therefore likely that effect of PA on the subsequent confounders in the time-updated analysis would not produce any major error in the time-updated analysis.

The major limitation with the present study is that it includes males only. Since the PA pattern is different in males and females [16], the present results have to be confirmed in women. Another limitation is that PA was self-reported, although we previously have shown PA to be associated with exercise performance and skeletal muscle fiber composition [15]. A more objective measure of PA, as could nowadays be assessed by accelerometry, might give other results, but it is reassuring that a recent report from the UK biobank reported a linear effect of accelerometry-derived PA on incident CVD during 5 years follow-up [17]. Thus, there will be some years before we have accelerometry-derived PA data over four decades. Until then, all results, including those presented in the present study, using self-reported PA should be taken with caution.

Strengths of our study design were the high participation rates, the repetitive use of identical measures at the four different examination cycles enabling the use of time-updated covariates for both PA and CVD risk factors, as well as 10-year follow-ups from four different ages within the same cohort and finally the ability to link these data to national health registries of high validity using the Swedish personal identifier number.

In conclusion, leisure-time physical activity is related to future CVD, mainly myocardial infarction and heart failure. This was most evident at 70 years of age. If a causal relationship between self-reported PA and CVD exists, this relationship might to a major degree be mediated by traditional risk factors.
